# Hospital Meetings, &c.

**Published:** 1901-02-09

**Authors:** 


### HOSPITAL MEETINGS, &c.

### GERMAN HOSPITAL.

The fifty-sixth annual meeting of the German Hospital was held at Winchester House, E.C., on Wednesday, January 30th, Mr. A. J. Allen presiding.

Before passing to the business of the meeting the chairman said he should like to say how deeply they all felt the loss of their beloved Queen, who had been the patron of the hospital since its foundation in 1845. The opening of the present reign showed how desirous Her Majesty's successor was to follow in the footsteps of his beloved mother, and from his record as Prince of Wales they might feel assured that the hospitals would lose nothing.

The report for the past year was then read by Mr. Gülich, the superintendent. It stated that the working of the hospital had been proceeding satisfactorily, with no marked changes to record. The in-patients numbered 1,631, as against 1,697 in 1899. This number included 209 cases of accidents—nearly all English—and 82 sanatorium patients; while 439 were admitted into the Convalescent Home. The out-patients, mostly English, numbered 19,365; while at the Eastern Dispensary there were treated 3,481, and at the Western Dispensary 1,551 cases. Ten beds had been placed at the disposal of the authorities for the use of soldiers from South Africa. Following the example of several English hospitals, with a view to the better control of the out-patient department, a lady almoner (Miss Ida M. Bush) had been appointed for one year on trial. This took place in December, so that no results could yet be reported. On December 11th the Kosher kitchen for Jewish in-patients was opened, and it was hoped this would prove a lasting boon to the Jewish community. It had been resolved to appoint an hon. assistant surgeon to see out-patients and to take the place of the visiting surgeons in case of leave of absence and illness, and the committee had conferred this post *ad interim* on Dr. Richard Wunsch, a former resident medical officer, subject to his qualifying himself for practice in this country. Dr. Wilhelm Scheffer and Dr. Felix Kraemer, the former resident medical officers, had returned to Germany, and had been succeeded by Dr. Otto Ihl from Kissingen, and Dr. Oscar Neuweiler from Aarau, Switzerland. It had also been thought expedient to change the lately created post of assistant resident medical officer to that of full resident medical officer. Dr. Dengler, who held this post, having resigned, Dr. F. Dammert had been appointed. The ordinary income for the year was £8,432, and the extraordinary (including £424 legacies, £2,000 for the endowment of two beds, £1,111 from the Kosher Kitchen Committee, and £250 special donation for the Prince of Wales's Fund. The ordinary expenditure was £9,580 and the extraordinary expenditure £3,476, of which £365 was for building improvements, £1,111 for the new Kosher kitchens and additional bedrooms, and £2,000 for the purchase of India Three per Cent. Stock for the endowments mentioned. The excess of expenditure over income was covered by a loan of £750 from the bankers. The committee expressed regret that annual subscriptions had fallen off, amounting to £1,403, as against £1,445 in 1899. The collection at the annual dinner amounted to £2,971.

The chairman, in briefly moving the adoption of the report, alluded to the death of the Baroness Schröder, the wife of their treasurer, which, coming so soon after the gift of £1,000 in commemoration of their golden wedding, made that entry in their report almost tragic. The committee, he said, thought it wise, under the present circumstances, not to hold the annual dinner this year. Under their constitution it should be held, but in the absence of any expressed objection they would take it as left to him to decide.

The report was seconded by Baron Deichmann and carried *nem. con.*, as were also votes of thanks to the president (the Duke of Cambridge) and the officers. The committee were re-elected, and the customary compliment to the chairman brought the proceedings to a close.

### GENERAL LYING-IN HOSPITAL.

The annual meeting of the Governors of the General Lying-in Hospital, York Road, Lambeth, was held on Wednesday, 30th ult., Lord Lister presiding. An address was adopted for presentation to H.M. the Queen Consort in which the Governors were glad to hope that the interest taken by the Throne for so many years might be continued. The annual report stated that 632 in-patients had been treated, and 1,786 out-patients. There was a slight decrease in the number of in-patients, measures having been taken, as suggested in the last medical report, to prevent overcrowding in the wards. The wives of soldiers always received special attention: it was regretted that the husbands of some of the patients had fallen in the early stages of the war. The Training School maintained its high reputation: during the year 83 nurses and 24 midwives had passed through it. The Committee were now able to realise more fully the benefits of the Convalescent Home at Woking. It was earnestly hoped that local taxation of hospitals, a growth of recent years, might speedily be abolished; a Bill would be presented to Parliament to relieve hospitals of this unjust imposition upon money given for work among the poor. The Hon. Treasurer said that the sum subscribed by H.M. Queen Victoria, in sixty-three annual subscriptions, amounted to £1,324.

Their thanks were due to the Duke of York, the Skinners' and Armourers' and Braziers' Companies, and other benefactors, to the usual donors of Christmas gifts, and to the nurses and midwives for a liberal contribution placed in the hands of the matron. The sum received for training fees was £1,405 19s.

The medical report stated that one death had occurred: this was a patient who had been unsuccessfully treated by her own doctor before admission; epilepsy had occurred after delivery; 777 women had been treated in their own homes. All the midwives trained during the year had passed the examination of the London Obstetrical Society.

In moving the adoption of the reports and balance sheet, Lord Lister said there was no doubt that the training of midwives was a most valuable function of the institution. It was very gratifying to see that the absence of septic disease and disease by communication continued; he felt particularly the value of sending out midwives, so that poor women were not left to the old style of terribly mischievous midwife. The poor were by this means better off than the middle classes, who preferred trusting to the mercies of the general practitioner, who had not hitherto learnt to trust to antiseptic precautions. The reports were adopted.

It was stated that the midwives belonging to a certain hospital conducted on vegetarian lines, finding the ground so admirably covered by the agents of this institution, had all departed.

The following appointments were made: President, Lord Lister; vice-presidents reappointed, with Sir John Williams in place of Dr. Grigg, deceased; Mr. Wm. Calder accountant; Messrs. G. G. Kennedy, H. N. Hamilton Hoare, and Dr. Louis S. Parkes members of Committee of Management.

A vote of thanks to Lord Lister ended the proceedings.

